# Rescue Blankets as Multifunctional Rescue Equipment in Alpine and Wilderness Emergencies—A Narrative Review and Clinical Implications

**DOI:** 10.3390/ijerph191912721

**Published:** 2022-10-05

**Authors:** Bernd Wallner, Hannah Salchner, Markus Isser, Thomas Schachner, Franz J. Wiedermann, Wolfgang Lederer

**Affiliations:** 1 Department of Anaesthesiology and Critical Care Medicine, Medical University of Innsbruck, Anichstr. 35, 6020 Innsbruck, Austria; 2 Austrian Mountain Rescue Service—Tyrol, Medical Division, Florianistr. 2, 6410 Telfs, Austria; 3 Department of Cardiac Surgery, Medical University of Innsbruck, Anichstr. 35, 6020 Innsbruck, Austria

**Keywords:** rescue blanket, hypothermia, emergency medicine, wilderness medicine, bandages, pelvic binder, tourniquet

## Abstract

The utilization of rescue blankets in pre-hospital emergency medicine exceeds protection from hypothermia and enhanced visibility by far. In this narrative review, we focus on emphasizing the alternative applications of these fascinating multifunctional tools in the pre-hospital setting. A literature search in PubMed^®^ and Web of Science^TM^ yielded 100 results (last update was on 8 July 2022), a total number of 26 of which were included in this narrative review. Nine articles assessing alternative functions of rescue blanket were further evaluated and described in more detail. In addition, we performed various experimental and observational trials to test the functionality of rescue practice in mountain emergency medicine. Newly fabricated rescue blankets proved to possess impressive robustness. We evaluated rescue blankets in their applicability to not only protect from hypothermia, but also as practical tools to treat catastrophic hemorrhage and bleeding limbs, to perform open pneumothorax chest seals in sucking chest wounds, to prevent damage to unprotected eyes on the glacier and as alternative instruments for transportation in the inaccessible areas. Rescue blankets are important rescue equipment in alpine and wilderness emergencies with multifunctional applications, and must be part of every personal medical kit.

## 1. Introduction

Rescue blankets are transparent polyethylene terephthalate foils coated with a thin aluminum layer [1]. The blankets, with their characteristic silver and gold surfaces, protect against hypothermia in out-of-hospital emergencies [2,3,4]. Rescue blankets have the ability to prevent and treat hypothermia by reducing heat loss through convection, conduction, evaporation, and thermal radiation [1]. The successful application of rescue blankets in the hospital setting, as well as by pre-hospital emergency medical services (EMS), underlines that blankets are compulsory emergency equipment [3,5]. A substantial benefit of rescue blankets is the low weight along with the low-bulk properties that require only little space.

Hypothermia is only one of many potential threats to mountaineers, sportsmen and EMS providers. Rescue blankets have several other properties, which make them multifunctional tools. The light reflection from the golden surface of the rescue blanket enhances visibility and therefore increases the probability of being found in search-and-rescue operations [6,7]. Another advantage is their impressively high tensile strength despite low weight, which enables alternative applications [8].

Through their broad applicability, rescue blankets have become an essential part of first aid kits and backpacks in mountain and wilderness environments. In this review, we aimed to evaluate the numerous applications of rescue blankets and to estimate the practicability in the treatment of emergencies by wilderness medicine and pre-hospital EMS. 

## 2. Materials and Methods

**Figure 1:**
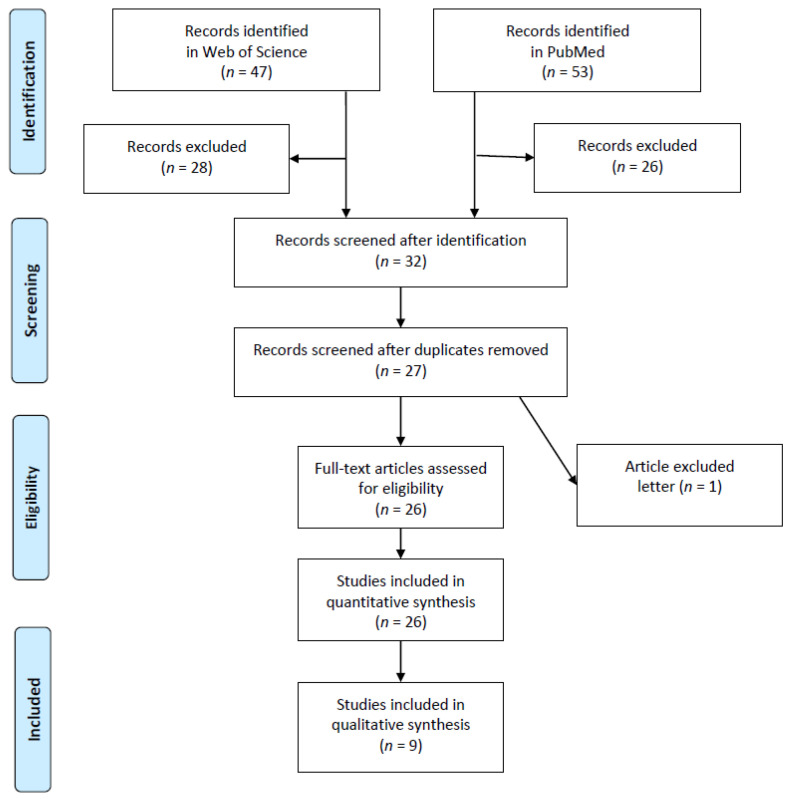
Literature research on rescue blankets and emergency medicine (PRISMA).

The method for identifying and including studies was consistent with the recommendations of the Cochrane Collaboration, as well as the guidelines of the Preferred Reporting Items for Systematic Reviews and Meta-Analyses Statement for Reporting Systematic Reviews (PRISMA) (Figure 1) [9]. This systematic search included literature from PubMed^®^ and the Web of Science^TM^, and considered studies published from 1990 until the 1 June 2022. The authors performed a broad and comprehensive search for articles and selected abstracts, titles and full texts using the following strategy: MeSh terms, i.e., “rescue blanket”, “survival blanket” and “space blanket” were combined with the search term of “emergency medicine” using both conjunctions, OR and AND (including text words and respective MeSh terms). Additionally, articles identified via manual search of the reference lists of included articles, as well as articles from grey literature (personal contacts of the authors), relevant to the topic. Screening on PubMed^®^ and Web of Science^TM^ for combined key words revealed the following: “survival blanket and emergency medicine” (28 in Web of Science^TM^ and 24 in PubMed^®^); “space blanket and emergency medicine” (4 in Web of Science^TM^ and 4 in PubMed^®^); “rescue blanket and emergency medicine” (15 in Web of Science^TM^ and 18 in PubMed^®^). After exclusion of papers that did not match the search criteria (in sequence of frequency: cooling blankets, warmed forced-air blankets, cotton blankets, rescue bags) and after removal of duplicates and a letter, 26 studies remained for evaluation (Figure 1). 

We further evaluated the use ofrescue blankets in various practical fields compared to similar applications found in the literature. Unfortunately, alternative specification of rescue blankets in the literature are scarce. We therefore had to rely on practical field tests.

## 3. Results

Beside the quantitative synthesis of the conventional function of rescue blankets to protect from hypothermia, wind and moisture, we qualitatively evaluated alternative functions of rescue blankets and identified nine articles for review [6,7,8,10,11,12,13,14,15]. The various alternative functions of rescue blankets comprised block of ultraviolet and infrared radiation, transparency, tensile strength, smoothness and tightness that allowed application as makeshifts of bandages, pelvic binders, tourniquets, chest seals, infection barriers, sunglasses and transport facilities in remote areas. A recent study further showed that rescue blankets could even provide a barrier during resuscitation and protect against pathogens transmitted by skin contact, aerosol or droplet infection [15].

In the following section, the alternative functions of rescue blankets were described and illustrated figuratively to be applied in wilderness and prehospital medicine. 

Table 1: List of the 26 articles included in the quantitative analysis of the narrative review. The grey lines represent those nine studies that indicate alternative applications of rescue blankets and were thus included in the qualitative analysis.

### 3.1. Alternative to the Arm-Sling or Triangular Bandage:

Injuries to the upper extremities are common when performing winter sports or leisure activities in mountains and wilderness environment. Rapid and safe immobilization does not only relief instant pain but also prevents secondary injuries to the limb.

Indications for an arm-sling include various injuries, fractures or dislocations involving shoulder, clavicle, humerus, forearm and hand [28]. The arm-sling was reported to prevent shoulder subluxation in stroke patients [29]. Traction and immobilization relieve pain. In case of clavicle fractures or dislocations of the shoulder, the arm-sling enables the patient to keep it in the most comfortable position. 

#### Application of a Rescue Blanket as Arm-Sling or Triangular Bandage

**Figure 2:**
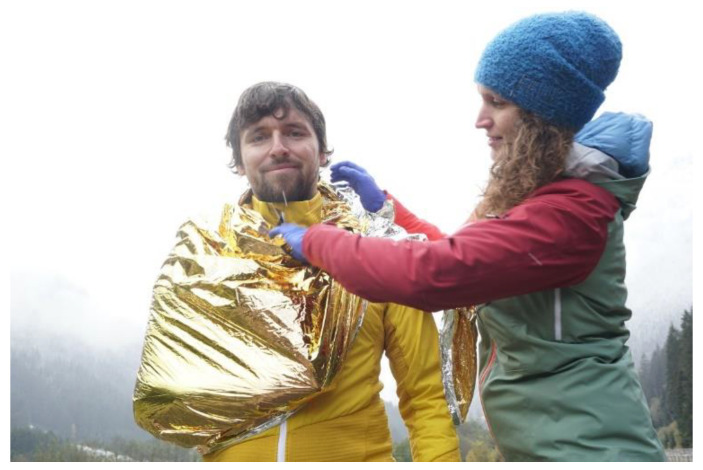
Application of a rescue blanket as arm-sling or triangular bandage.

Due to the high tensile strength rescue blankets can serve similar tasks as triangular bandages [8]. For makeshift triangular bandaging the longitudinally unfolded rescue blanket is applied below the injured forearm like a tie, with the two endings knotted to ring behind the neck. When using the blanket to support the elbow, the fanned out rescue blanket is applied around the elbow with the two endings tied to a ring behind the neck (Figure 2).

### 3.2. Alternative to the Figure-of-Eight (Rucksack Bandage):

A figure-of-eight bandage is used to disconnect the overlying fragments of a fractured clavicle. Furthermore, the figure-of-eight bandage stabilizes and immobilizes the shoulder joint through pulling both shoulders backwards. This forces the patient into a straight-backed posture. When the fragments of the fractured clavicle are separated by traction, pain is relieved instantly. Andersen et al. observed that patient satisfaction was lower with the figure-of-eight bandage [30]. Healing was not affected, and functional outcome did not differ between the two types of immobilizations. The authors prefer the broad arm sling to the figure-of-eight bandage because of the rapid reduction of pain, the ease of handling and the more efficient immobilization during transport in the pre-hospital setting [31]. 

#### Application of a Rescue Blanket as Figure-of-Eight (Rucksack Bandage):

**Figure 3:**
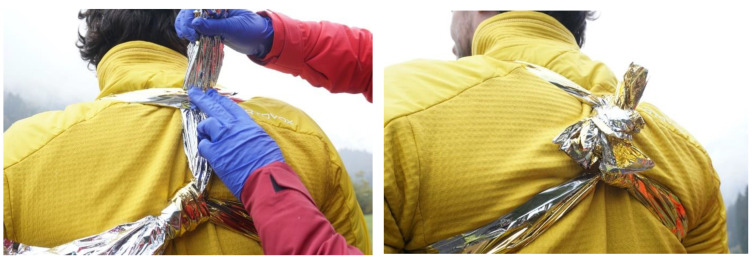
Application of a rescue blanket as figure-of-eight.

The high tensile strength of rescue blankets allows them to be used as figure-of-eight bandage [8]. During application of the rescue blankets with the patient in sitting position, the blanket is arranged to pull back both shoulders and form a figure-of-eight on the back of the patient (Figure 3). 

After application, the bandage must be checked regularly for swelling or temporary numbness which can result from compression of nerves and blood vessels. Depending on the type of fracture and region involved, immobilization can be achieved without aids or with a backpack bandage, a gilet, an arm sling or a Gilchrist bandage. The latter is mainly used for fractures of the lateral collarbone.

### 3.3. Alternative to the Pelvic Sling/Pelvic Binder 

A pelvic sling is used in pre-hospital emergency medicine to establish circumferential compression of a fractured hip. The prime example for the use of pelvic slings is the open book injury, which has a high risk of massive blood loss. The aim of the pelvic sling is to diminish bleeding by reducing the space between the bone fragments. The pelvic sling is established to stabilize and immobilize the hip. It also reduces pain and facilitates the transport of the patient. Applying a pelvic sling should be considered in these circumstances, especially in hemodynamically unstable patients. An isolated femoral neck fractures would be one of the few contraindications. The various commercial products on the market are mostly suitable for professional rescuers, rather than for sportsmen engaging in mountaineering or travelling during leisure activities. Newly fabricated rescue blankets are safe, strong and easy-to-use alternatives to commercial products.

#### Application of a Rescue Blanket as Pelvic Sling/Pelvic Binder

Four “P words” to remember the requirements for creating a pelvic sling:

- Pocket: emptying pockets in the hip region prior to application to avoid injury from objects such as a key chain, lighter or mobile phone
- Penis: ensuring that no harm arises from pressure after application
- Pulse: pulse control of the lower extremities after application

**Figure 4:**
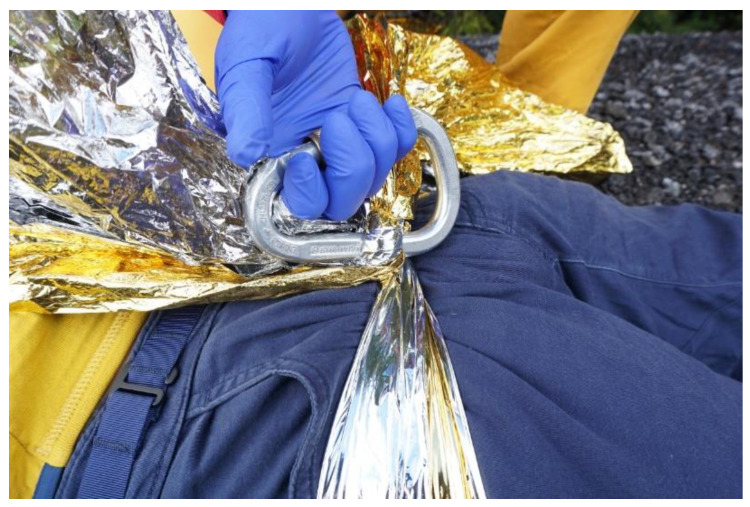
Application of a rescue blanket as pelvic sling/pelvic binder.

Positioning: the sling should be positioned over the greater trochanters The rescue blanket is placed under the patient’s hip at the height of the greater trochanter with the two endings knotted on the front and tightened e.g., by using a carabiner (Figure 4). With the patient in supine position, the rescue blanket is first drawn through below the knees and then moved below the hip. Adjustment with jerky “saw-like” movements is not generally recommended. Inward rotation of the legs and fixation at knee level may further improve the splinting effect.

### 3.4. Alternative to the Tourniquet

Severe extremity hemorrhage can be controlled by compression and application of tourniquets in prehospital emergencies [32,33].

To achieve an adequate occlusion pressure is mandatory. Improvised tourniquets are less reliable than well-designed tourniquets. If placed only tight enough to occlude venous return, they can even increase blood loss [34]. Potential complications include nerve palsy, compartment syndrome, venous thromboembolic events, and post-ischemic reperfusion damage [35]. Pain from compression was observed to be less common with rescue blankets used as makeshift tourniquets [14]. Whenever rescuers run out of commercial tourniquets in mass casualties and incidents with multiple severely bleeding victims rescue blankets may serve as improvised tourniquets [14].

#### Application of a Rescue Blanket as Tourniquet

**Figure 5:**
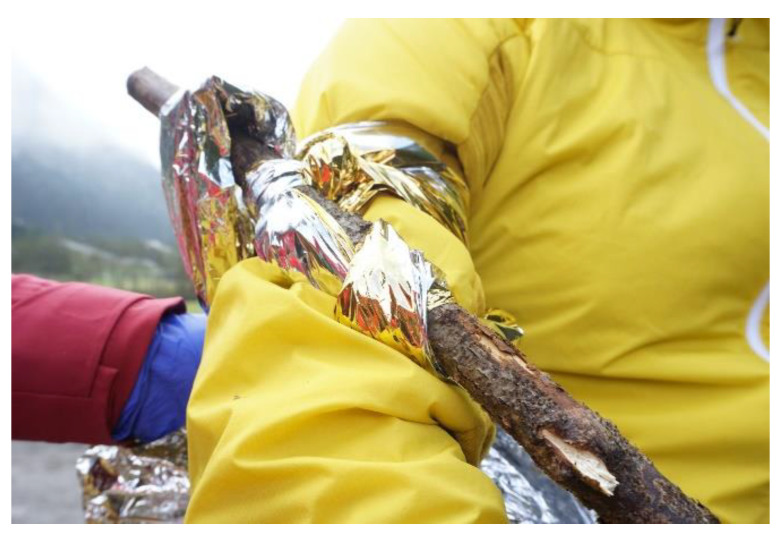
Application of a rescue blanket as tourniquet.

Similar to commercial tourniquets, rescue blankets are positioned close to the trunk, e.g., in upper limb injuries within 5 cm of the armpit. A 7 cm broad segment of a longitudinally unfolded rescue blanket is routed twice around the limb like a scarf. The two endings of the SB are joined with two common square knots. Then a windlass, e.g., a pair of scissors or a rod, is inserted between the knots. Torque is applied by twisting the windlass until the bleeding has stopped. (Figure 5). Then the windlass is tucked under a wrap and secured with the two endings of the rescue blanket.

Survival blankets can be used appropriately as makeshift tourniquets, but incomplete occlusion of the radial artery was reported in almost half of the applications when a carabiner was used as makeshift windlass [14]. This underlines the need of repeated bleeding control during maintenance of tourniquet pressure, irrespective of the used device.

### 3.5. Alternative to Transportation Tools in Wilderness Emergencies

Terrestrial transport of injured and ill patients can be a strain to the patients and strenuous for the rescuers. Occasionally, the emergency patient has to be rapidly rescued from a dangerous area). Unless there is imminent danger, the patient’s health conditions need to be stabile prior to transport until:

- Vital functions are within normal limits
- Life-threatening bleeding was stopped
- There is no risk of shock

#### Application of a Rescue Blanket as Transportation Tool

One rescuer having grasped the hand of another rescuer can transport a conscious patient sitting on their lower arms over short distances. A triangular bandage or a scarf can facilitate transport when the endings are joined with a knot to a ring. This creates kind of a seat on which the injured person can then be carried. A longitudinally unfolded rescue blanket with the two endings knotted to a ring is strong enough to carry adult persons.

**Figure 6:**
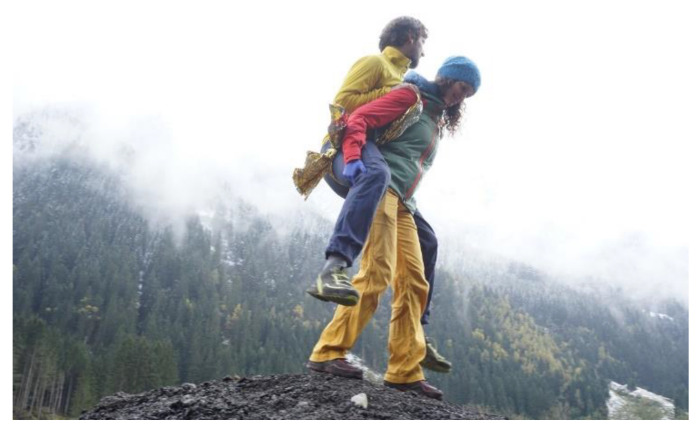
Application of a rescue blanket as transportation tool.

- Two rescuers standing next to each other reach into the carrying ring from above with their outer hands. The “inner arms” grasp the shoulder of the next man and form a backrest. The patient in sitting position embraces the wearer’s shoulders with his arms.
- With two rings, one around each thigh, the patient can be carried in sitting position on the back of a single rescuer (Figure 6).
- So far, we cannot tell if two rescuers can transport an unconscious person using a hammock made of two completely unfolded rescue blankets.

### 3.6. Electromagnetic Radiation and Eye Protection

Although the silver side of the blanket is highly reflective and flashes in the sun, but the gold side facing up increases a victim’s visibility even more in a snow and glacier environment. Furthermore, rescue blankets can effectively block infrared radiation and hamper thermal imaging in search and rescue missions [6].

Injury to eyes and skin may arise from high-energy rays in the ultraviolet (UV) spectrum and high-energy visible (HEV) light in the violet/blue band during mountain hikes. Painful injuries to the cornea and conjunctiva are well-known medical emergencies resulting from intense solar radiation reflected by snow or glaciers. Increased probability of suffering from ocular damage from lasting solar radiation may occur, despite wearing snow goggles, if high-energy rays from the side and from around the frame can reach the eye and cause snow blindness and retinal burn. The International Commission for Mountain Emergency Medicine (ICAR-MEDCOM) recommends that patients with any form of sight-threatening eye problem or unexplained visual loss at high altitudes should descend immediately. However, in some cases of acute pain and temporary loss of vision descent may turn out to be impossible.

#### Application of a Rescue Blanket as Emergency Eye Protection

**Figure 7:**
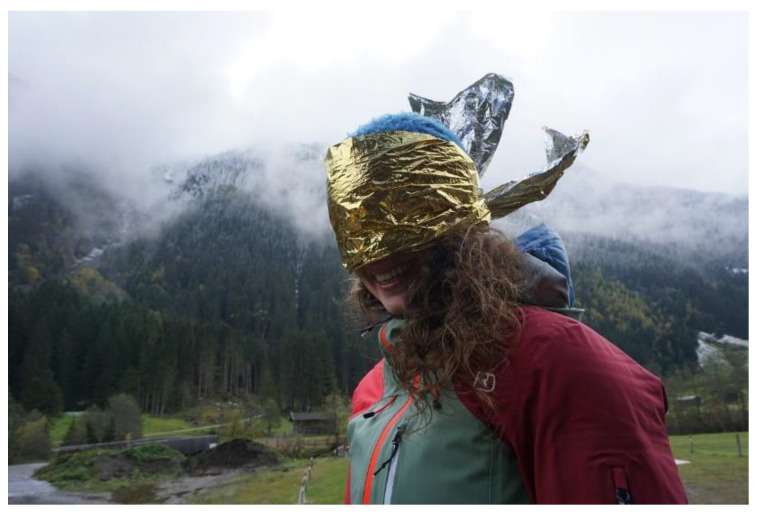
Application of a rescue blanket as emergency eye protection.

Contrasting qualities are well known for multi-functional rescue blankets. In an experimental investigation performed by Isser M. et al. single-layer rescue blankets were shown to be sufficiently permeable for visible light [7]. In addition, they adequately blocked transmission for UV rays and HEV light in the violet/blue band to protect from solar radiation. Transmission for ultraviolet B rays afforded by each tested rescue sheet brand was between 0% and 1% for the single layer. As there is still sufficient transparency for visible light, a single-layer rescue sheet put over the mountaineer’s head allows him to descend under his own power while being protected from ultraviolet radiation comparable to conventional sunglasses. However, this does not apply to the military edition of the recue blanket that is covered with an additional layer of green color (Figure 7).

### 3.7. Alternative to the Chest Seal in Perforating Chest Trauma

Perforating chest wounds from civil and military casualties may cause an open pneumothorax. If air gets trapped within the pleural space, a life-threatening tension pneumothorax may arise. Compression of large intrathoracic vessels and shift of the heart to the initially unimpaired lung may lead to fast hemodynamic deterioration. In the prehospital setting a non-occlusive chest seal is essential treatment for a sucking chest wound according to the ERC guidelines [36]. However, if a non-occlusive dressing is not available, a makeshift chest seal of polyvinyl chloride packing may be used by first aid providers [36].

#### Application of a Rescue Blanket as Makeshift Chest Seal

**Figure 8:**
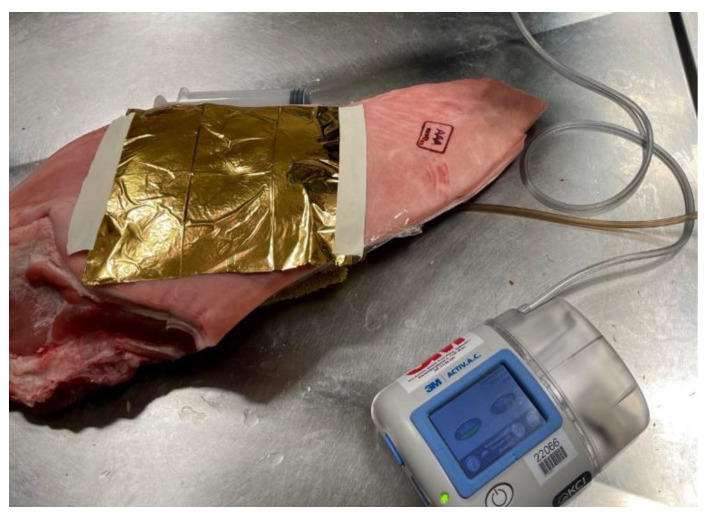
Application of a rescue blanket as a makeshift chest seal.

Schachner et al. recently developed an *ex vivo* porcine model of open pneumothorax, and observed favorable properties of the rescue blanket when used as a non-occlusive chest seal (Figure 8) [12]. Application of a loosely fixed wet-surfaced piece of rescue blanket proved to have good sealing effects in treating an experimental sucking chest wound. Then again, air could evade the cover easily under positive intrathoracic pressure. Since commercially available chest seals are uncommon in first aid kits of those who participate in outdoor sport, one single piece of the rescue blanket might serve as a valuable substitute [12].

### 3.8. Body-Shield Resuscitation Barrier Device to Protect from Blood

Rescuers and first-aid providers without protection are exposed to abundant pathogens while performing cardiopulmonary resuscitation (CPR), since they may get in direct contact with either the patient’s blood, other body fluids or aerosols [11,13]. The fear of contracting a disease has become the prime reason for lay rescuers and first-aid providers to refrain from performing CPR [15]. During the corona virus disease (COVID-19) pandemic this fear has further increased, therefore the European Resuscitation Council (ERC) omitted recommendations for ventilation attempts by lay rescuers and recommending compression-only CPR for the duration of the COVID-19 pandemic [11,37].

The study by Hermann et al. compares four different foils regarding their potential to reduce the rescuers exposure to blood. As the quantity of exposed blood differs within the run-off figure of a blood stain, the likelihood of infection depends on direct contact with either blood drops or large run-off areas [15].

This experimental study shows that, on the one hand, delayed clotting on different foil surfaces goes along with larger areas of contamination, which increases the chance of coming into contact with potential germs [15]. On the other hand, the lesser content of germs per area decreases the likelihood of contracting an infection [15]. Therefore, blanketing the patient with a foil can protect against pathogens transmitted by skin contact, aerosol or droplet infection by reducing the contamination by blood, vomit and secretions [11,13,15].

## 4. Conclusions

In conclusion, we can state that rescue blankets are indispensable, low-weight pieces of equipment in emergency kits. Their physical properties and wide range of applications exceed mere protection from hypothermia through insulation by a substantial measure. In this narrative review, we highlight a variety of alternative applications of rescue blankets and performed experimental and observational trials to test the functionality of rescue practice in mountain emergency medicine. Newly fabricated rescue blankets demonstrate impressive robustness. We systematically reviewed the application of rescue blankets as alternatives to triangular arm-slings, to figure-of-eight bandages, as makeshift pelvic binder, as tourniquet in hemorrhage emergencies and as a transportation tool for emergencies in remote areas. Rescue blankets proved to be effective makeshift chest seals in sucking chest wounds, eye protections from UV-radiation, as well as body-shield resuscitation barrier devices.

## 5. Outlook

The potential of rescue blankets is not yet exhausted, and various alternative applications warrant exploration. The electrical conductivity of rescue blankets from defibrillation during advanced life support and in the case of lightning stroke remains obscure. In addition, the application of rescue blanket to non-adherent metalized bandage of open wounds and burns remains to be investigated. These are only excerpts from a long list of potential application possibilities of this fascinating tool.

## Figures and Tables

**Table 1:** Findings of the reviewed sources after screening on PubMed^®^ and Web of Science^TM^.

Authors	Objective	Type of Source	Study Design	Major Research Topic
Schultz et al. (2003) [16]	Emergency treatment after earthquake	Medical record	Retrospective data questionnaire	Emergency care
Henriksson et al. (2012) [17]	Wet clothing removal and vapor barrier	Manikin in thermal chamber	Experimental	Hypothermia
Karlsen et al. (2013) [18]	Prehospital management of hypothermia prevention	Prehospital medical service evaluation	Structured telephone interviews	Hypothermia
Jussila et al. (2014) [19]	Maritime prehospital transportation and thermal protective properties	Thermal manikin and human study	Experimental and clinical	Hypothermia, wind and moisture
Henriksson (2015) [5]	Wet clothing removal and vapor barrier	Volunteers in thermal chamber	Experimental	Hypothermia
Perlman et al. (2016) [20]	Temperature monitoring and hypothermia prevention	Physiological effects	Systematic review in trauma patients	Hypothermia
Zasa et al. (2016) [21]	Thermal protective properties of different casualty coverings	Human torso model	Experimental	Hypothermia and rewarming
Paal et al. (2016) [22]	Management and outcome of accidental hypothermia	Physiological effects	Systematic review	Hypothermia
Haverkamp et al. (2017) [2]	Prehospital management of hypothermia prevention	Literature research	Clinical and experimental	Hypothermia
Henriksson et al. (2017) [4]	Equipment for prevention of accidental hypothermia	Prehospital service evaluation	Questionnaire	Hypothermia
Freeman et al. (2018) [3]	Equipment for prevention of accidental hypothermia	Prehospital service evaluation	Questionnaire	Hypothermia
Stroop et al. (2019) [23]	Accident-related hypothermia and rewarming strategies	Volunteer in extrication operations in three scenarios	Retrospective data, experimental	Hypothermia
Isser et al. (2019) [7]	UV radial protective properties of two brands	Optometrical measurement by lens analyzer	Experimental	UV radiation and transparency
Phillips et al. (2019) [24]	Thermal protective properties	Case study	Clinical	Hypothermia
Kranebitter et al. (2020) [10]	Reflectivity of electromagnetic radiation	Thermal imaging	Experimental and field study	Thermoradiation
Barcala-Fureolos et al. (2020) [11]	Blanketing of emergency patients	Transmission control	Pilot study	Barrier CPR
Isser at al. (2020) [6]	Infrared radiation and visibility in search and rescue missions	Thermal imaging	Experimental field study	Thermoradiation
Isser at al. (2020) [8]	Tensile strength of two brands	Tensile strength testing device	Experimental	Haemorrhage control
Schachner et al. (2021) [12]	Open pneumothorax	Ex vivo porcine model	Experimental	Haemorrhage control
Barcala-Fureolos et al. (2021) [13]	Personal protective equipment comparison of equipment	Transmission control	Pilot study	Barrier CPR
Stroop et al. (2021) [25]	Accident-related hypothermia	Volunteer in four simulated scenarios of traffic accidents	Self-experimental	Hypothermia
Dvir et al. (2021) [26]	Thermal protective properties of different casualty coverings	Human torso model	Experimental	Hypothermia, rewarming
Lønnee et al. (2021) [27]	Medical supplies and treatment at a major music festival	Medical record	Retrospective	Emergency care, hypothermia
Salchner et al. (2022) [14]	Makeshift tourniquet	Volunteers in cross-over	Experimental	Haemorrhage control
Kosiński et al. (2022) [1]	Thermal protective properties incl. water, wind and radiation	Literature research	Systematic review	Hypothermia, wind and moisture
Hermann et al. (2022) [15]	Body-shield resuscitation barrier	Evaluation of surface properties	Experimental	Barrier CPR

The lines colored in GREY highlight those articles that were included in the quantitative analysis.

## Data Availability

The datasets used and analyzed during the current study are available from the corresponding author (B.W. bernd.wallner@i-med.ac.at) on reasonable request.
